# Mitogenomics Provide New Phylogenetic Insights of the Family Apataniidae (Trichoptera: Integripalpia)

**DOI:** 10.3390/insects15120973

**Published:** 2024-12-06

**Authors:** Xinyu Ge, Jingyuan Wang, Haoming Zang, Lu Chai, Wenbin Liu, Jiwei Zhang, Chuncai Yan, Beixin Wang

**Affiliations:** 1 Tianjin Key Laboratory of Conservation and Utilization of Animal Diversity, College of Life Sciences, Tianjin Normal University, Tianjin 300387, China; skygxy@tjnu.edu.cn (X.G.); 2410170016@stu.tjnu.edu.cn (L.C.); skylwb@tjnu.edu.cn (W.L.); 2 Department of Entomology, College of Plant Protection, Nanjing Agricultural University, Nanjing 210095, Chinawangbeixin@njau.edu.cn (B.W.); 3 Changjiang Basin Ecology and Environment Monitoring and Scientific Research Center, Changjiang Basin Ecology and Environment Administration, Ministry of Ecology and Environment, Wuhan 430010, China

**Keywords:** mitogenome, phylogeny, *Apatania*, *Apatidelia*, *Apataniana*

## Abstract

The family Apataniidae consists of two subfamilies, Apataniinae and Moropsychinae. Currently, there are 204 valid species of Apataniidae, which are widely distributed throughout the northern hemisphere. The larvae typically inhabit cold-water environments, and they serve as biological indicators for monitoring the health of freshwater ecosystems. The phylogenetic relationships within Apataniidae are not fully understood. Moreover, the available molecular data of Apataniidae are still limited. Herein, we provided the mitochondrial genomes of eight apataniid species and compared them with the published mitochondrial genomes of Apataniidae. The nine newly obtained sequences ranged from 15,070 bp to 16,737 bp in length. The results of the nonsynonymous with synonymous substitution rates displayed that *ATP8* had the highest evolutionary rate, while *COXI* exhibited the lowest. The *ND4L* may be an effective molecular marker for the classification of the Apataniidae. Based on the published mitogenomes, we constructed a phylogenetic tree for Limnephiloidea and conducted a preliminary analysis of its advanced phylogeny. The ML and BI analyses recover the monophyly of Apataniidae and Limnephilidae. Except for PCG, BI tree based on other matrices consistently showed the topology: (*Apataniana* + (*Moropsyche* + (*Apatidelia* + *Apatania*))). The taxonomic status of *Apatania* and *Apatidelia* were also preliminarily explored. The mitochondrial genome of Apataniidae provides critical genomic resources for understanding the phylogenetic relationships of Apataniidae.

## Simple Summary

The phylogenetic relationships of Apataniidae are not fully understood. In addition, molecular data for phylogeny within the family Apataniidae are seriously insufficient, and phylogenetic studies within the family Apataniidae are very rare. The mitochondrial genome stands as an effective marker for elucidating phylogenetic relationships. In this paper, mitochondrial genomes of eight Apataniidae and one Limnephilidae were provided. We analyzed the base composition, codon usage, and evolutionary rate of the apataniid mitogenome, expanding our understanding of the mitogenome of the Apataniidae. Then, the phylogenetic relationship of the Apataniidae and subfamily level were reestablished based on the mitogenomes. The results of this study provide a basis for further studies on the phylogeny and evolution of Apataniidae from a mitogenomic perspective.

## 1. Introduction

The family Apataniidae belongs to the superfamily Limnephiloidea (Trichoptera: Integripalpia) and comprises 18 genera, eight of which are monotypic [1]. It consists of two subfamilies, Apataniinae Wallengren, 1886 and Moropsychinae Schmid, 1953. Currently, there are 204 valid species of Apataniidae, which are widely distributed throughout the northern hemisphere [2]. Adults are either univoltine or bivoltine, and relatively small in size. Diapause has also been observed in the life history of some apataniid species, such as the genus *Allomyia*, which inhabits intermittently arid mountain streams [3]. The larvae construct slightly tapered cases made of small rock fragments and typically inhabit cold-water environments, such as cool streams and rivers [4]. However, in the far north or at high elevations, the genus *Apatania* larvae can be found in glacial lakes [5]. Some species feed on fine particles of organic matter suspended in the water, while others are predatory. Additionally, in North America and Japan, five species of the genus *Manophylax* larvae have been reported to thrive on terrestrial rock surfaces, where they are most active and feed on algae and moss [6]. Its larvae and pupae are found in water sources, such as streams and lakes, and are sensitive to water quality. As a result, they serve as biological indicators for monitoring the health of freshwater ecosystems [7].

MacLachlan divided the family Limnephilidae into two sections based on whether the fore-wing Sc venation terminates at the posterior margin [8]. Section I includes *Limnephilus*, Leach, 1815, *Grammotaulius*, Kolenati, 1848, *Anabolia* Stephens, 1837, and so on. Section II includes *Apatania* Kolenati, 1848 and *Radema* Hagen 1864. Wallengren erected the family Apataniidae with *Apatania* Curtis, 1834 as the type genus by original designation. Subsequently, many scholars have used morphological characteristics to explore the taxonomic status of the family Apataniidae. The results classified the family Apataniidae as a tribe or a subfamily of the family Limnephilidae until 1992 [9,10]. Gall combined Apataniinae and the four independent genera (*Allomyia* Banks, 1916, *Manophylax* Wiggins, 1973, *Moselyana* Denning, 1949, and *Pedomoecus* Ross, 1947) to establish the family Apataniidae [4]. This classification is still valid today.

At present, there are still challenges in accurately identifying species and determining phylogenetic relationships among some genera within Apataniidae. Previous studies on this family have primarily focused on species identification and genus reviews based on morphology in specific regions, such as Lake Baikal and Japan [11,12,13,14]. In addition, some researchers have combined morphological analysis with DNA barcoding to study larval descriptions and the biogeography of apataniid species [15,16,17,18]. However, molecular data for phylogenetic analysis within the family Apataniidae are severely lacking, and phylogenetic studies within this family are quite rare.

The mitochondrial genome (mitogenome) stands as an effective marker for elucidating phylogenetic relationships and evolutionary history of insect groups, characterized by its unique features of low sequence recombination, maternal inheritance, and a fast-evolutionary rate [19,20]. Typically, the mitogenome contains 37 genes, including 13 protein-coding genes (PCGs), two ribosomal RNA genes, and a control region [21]. Wang et al. reported the first mitochondrial genome of the moth, but it was incomplete (missing *s-rRNA* and CR) [22]. With the advancement of high-throughput sequencing technology, the number of Trichoptera mitogenomes sequenced is increasing at an extremely rapid rate [21,23,24]. In the past two years, over 100 new mitogenomes of Trichoptera have been reported, greatly enhancing our understanding of mitochondrial genome structure and Trichoptera evolution [23]. In the suborder Annulipalpia, a large number of gene rearrangements (PCGs, tRNA, and rRNA) in the mitogenome, combined with a high evolutionary rate, has hindered the mitogenome’s ability to accurately resolve phylogenetic relationships within each family [21]. Additionally, the nucleotide substitution rate of each gene is low in Limnephiloidea, providing an opportunity to elucidate the phylogeny of this family based on mitogenome data [21]. However, compared to the many mitochondrial genomes published in the family Limnephilidae, the mitochondrial genomes of only one apataniid species have been published, which greatly limits our understanding of the evolution of the family Apataniidae [23]. Moreover, comparative analyses of nucleotide composition and evolutionary rates among Apataniidae have not been conducted.

In this study, mitogenome sequencing was assembled, with annotation on eight species of Apataniidae and one of Limnephilidae. The mitogenomes of three genera are reported here for the first time. We analyzed the genome structure, base composition, codon usage, and evolutionary rate of the apataniid mitogenome, thereby expanding our understanding of the Apataniidae mitogenome. Subsequently, we combined the published mitogenome from within Limnephiloidea, and performed a systematic genome analysis of the family using ML model and GAT + GTR model analysis and to explore the phylogenetic location of the family and within the family. The results of this study provide new insights into the phylogeny and evolution of Apataniidae from a mitogenomic perspective. Additionally, as the apataniid species are primarily distributed in the middle and high latitude drainage basins, these new mitogenomes offer valuable reference data for studying aquatic biodiversity in northern streams using multi-marker DNA metabarcoding techniques.

## 2. Materials and Methods

### 2.1. Sampling Collection and DNA Extraction

A total of eight adult specimens of apataniid species were collected from Qinghai, Guanxi, and Xinjiang province. All specimens were collected during 2019–2024 with 15 w ultraviolet light sheet traps at night. Detailed information is shown in Table S1. Then, specimen identifications using morphological examination followed [25]. The specimens were then sorted and stored in 100% alcohol at −20 °C temperature until used for DNA extraction. The voucher specimen was deposited in the College of Life Science and Technology, Tianjin normal University, Tianjin, China. Total genomic DNA was extracted from the legs using the animal tissue protocol provided by the TIANamp Genomic DNA Kit (DP304; TIANGEN, Beijing, China), following the manufacturer’s instructions.

### 2.2. Sequence Assembly and Annotation

The *mtCOI* barcoding (685 bp) was obtained and analyzed following the procedures of Ge et al. [23]. The library was constructed with an insert size of 350 bp. The Illumina NovaSeq xplus platform (United States Illumina Company, San Diego, CA, USA) and the DNBSEQ-T7 platform (Beijing Genomics Institution, Beijing, China) were used to generate sequencing in PE-150 bp model, and then approximately 6 Gb clean data were produced for subsequent analysis. The raw data of *Drusus annulatus* (Stephens, 1837) were downloaded from Sequence Read Archive (SRA). NOVOPlasty v3.8.3 (Brussel, Belgium) [26] was used to assemble nine mitogenome with *mtCOI* sequences as seeds and k-mer sizes of 39 bp. The MITOS2 webserver [27] was used to predict tRNAs and their secondary structure and positions, employing the invertebrate mitochondrial genetic code. Furthermore, the positions of 13 PCGs were identified using Geneious v2024.0.5 (Boston, MA, USA), through the identification of open reading frames (ORFs) that refer to the invertebrate mitochondrial genetic codon. The boundary between PCGs and rRNA was manually corrected by comparing reference sequences. All new sequenced mitogenome were submitted to GenBank (for accession number).

### 2.3. Composition Analyses, RSCU, and Evolutionary Rate

SeqKit v0.16.0 (Chongqing, China) [28] was used calculate the nucleotide composition and bias of each gene and CR. AT-skew and GC-skew were calculated by two formulas: AT-skew = (A − T)/(A + T), GC-skew = (G − C)/(G + C). DnaSP v6.0 (Barcelona, Spain) [29] was used to calculate the rates of non-synonymous substitution rate (Ka)/synonymous substitution rate (Ks) for each PCG. It was also used to assess the nucleotide diversity of the 13 PCGs of the eight apataniid species. The relative synonymous codon usage (RSCU) of eight species of Apataniidae were calculated using MEGA X (Philadelphia, PA, USA), and R v4.0.3 [30] was employed for visualization. CG-view (https://cgview.ca/, accessed on 8 August 2024), an online server, was used to generate the visual sequence features of the mitogenome.

### 2.4. Phylogenetic Analyses

In this study, we analyzed eight apataniid species of the one limnephilid species. In addition, the mitogenomes of 10 species of were download from GenBank for the phylogenetic and comparative mitogenomic analyses. We selected three species, including *Goera horni* Navas, 1926, *Goera fissa* Ulmer, 1926, and *Uenoa lobata* (Hwang, 1957) (Uenoidae Iwata, 1927) that are closely related to Limnephiloidea as outgroups. Detailed taxon information was shown in Table S2. The 13 PCGs and two rRNAs genes of 22 mitogenomes were used conducted phylogenetic relationship of Limnephiloidea.

The nucleotide and protein sequences for each entity were subjected to alignment procedures using MAFFT v7.470 (Osaka, Japan) [31] with the L-INS-I algorithm. Subsequently, these aligned sequences underwent trimming using trimal v1.4.1 (Barcelona, Spain) [32], with the application of the “-automated1” strategy to remove redundant or noisy data. Following trimming, the resulting alignments were systematically concatenated, as five supermatrices were prepared for phylogenetic analyses using FASconCAT-G v1.04 (Bonn, Germany) [33]. The results include: (1) except the third codon position of 13 PCGs nucleotide sequences (PCG12 matrix); (2) all positions of 13 PCGs nucleotide sequences (PCG matrix); (3) except the third codon position of 13 PCGs and two rRNAs nucleotide sequences (PCG12R matrix); (4) all positions of 13 PCGs and two rRNAs nucleotide sequences (PCG123R matrix); (5) all PCGs of amino acid reads (PCGAA matrix). We conducted a preliminary analysis of the heterogeneity in sequence divergence within five supermatrices, employing ALIGROOVE v1.0.7 [34] (Bonn, Germany) and utilizing its default sliding window size for this purpose. The nucleotide was treated as an ambiguity substitution model, and the amino acid matrix with the BLOSUM62 model. The substitution saturation of the first, second, and third codon position of each gene was assessed using DAMBE v.7.2.32 (Ottawa, ON, Canada) [35].

Each supermatrix was utilized to infer phylogenetic relationships through two distinct methods: Bayesian Inference (BI) and Maximum Likelihood (ML). For the ML analysis, the optimal substitution models for each gene partition were selected using MODELFINDER [36], integrated within IQ-TREE v2.2.2.7 (Canberra, ACT, Australia) [37]. To mitigate the impact of heterogeneity, we applied the Posterior Mean Site Frequency (PMSF) model’s specific strategies to reduce the heterogeneous effect for amino acid. The bootstrapping phase and node support was calculated by using 1000 UFBoot2 (-alrt 1000) [38] and 1000 SH-aLRT (-B 1000) replicates [38] in all ML analyses. The BI tree was conducted using Phylobayes-MPI v1.8 (Montréal, QC, Canada) [39], with the site-heterogeneous mixture model CAT + GTR. The initial 25% trees of two Markov chain Monte Carlo chains (MCMC) runs were discarded as burn-in, and then stopped after the runs had satisfactorily converged. Finally, a consensus tree was subsequently computed, and displayed using Figtree v1.4.3 [40].

## 3. Results

### 3.1. Mitogenomic Organization in Apataniidae

**Figure 1:**
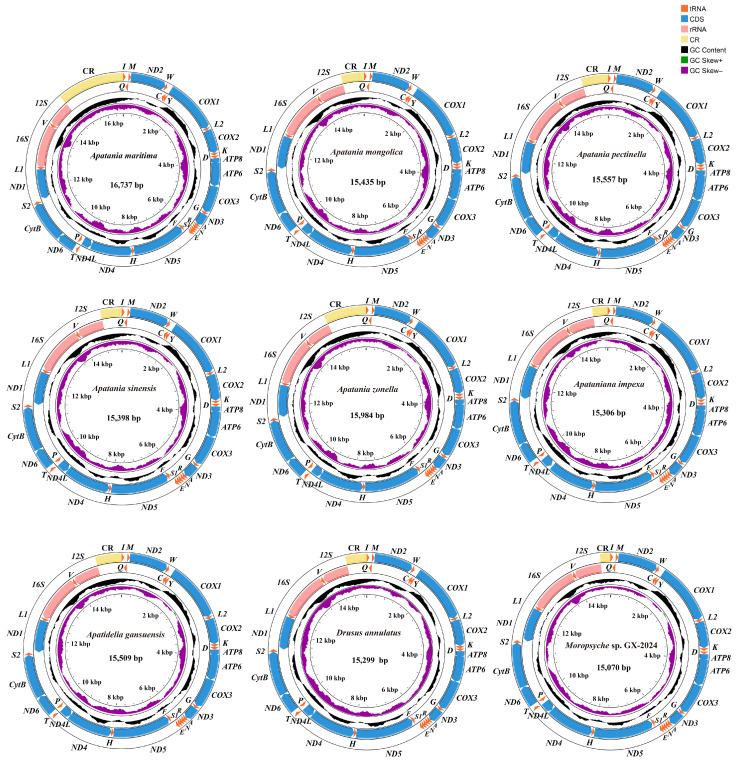
The mitogenome map depicted the nine species mitogenome of Apataniidae and Limnephilidae. The arrow served as a guide, pointing to the orientation of gene transcription. We used standardized abbreviations to denote PCGs and rRNAs, while single-letter abbreviations were chosen for tRNAs. The second circle highlighted the GC content of the entire mitogenome, whereas the third circle revealed the GC-skew. The innermost circle encapsulated the length of the entire mitogenome, providing a comprehensive visualization of its characteristics.

Approximately 6 Gb clean data were produced for each sample. A total of nine newly mitogenomes of were obtained in this study, which were *Apatania pectinella* Mey and Yang, 2001; *Apatania mongolica* Martynov, 1914; *Apatania maritima* Ivanov and Levanidova, 1993; *Apatania sinensis* Martynov, 1914; *Apatania zonella* Zetterstedt, 1840; *Apataniana impexa* Schmid, 1968; *Apatidelia gansuensis* Mey, 1997; *Moropsyche* sp. GX-2024; and *Drusus annulatus* (Figure 1). Each mitogenome of the species contains 37 typical genes (13 PCGs, 2 rRNAs, and 22 tRNAs) and 1 CR, which is usually present in most insect mitogenomes (Figure 1). Among them, the CR of *Apatania pectinella* and *Apatania maritima* failed to complete sequencing due to the high A + T content. The nine newly obtained sequences ranged in length from 15,070 bp (*Apatania maritima*) to 16,737 bp (*Moropsyche* sp. GX-2024). The lengths of 13 PCGs tRNA, *l-rRNA*, and *s-rRNA* were not obviously different, ranging from 11,187 bp to 11,220 bp, 1444 to 1464 bp, 1364 to 1389 bp, and 778 to 787 bp, respectively (Table S3).

### 3.2. Nucleotide Composition

The significant variation in the mitogenome length of each species mainly occurs in the CR (ranging from 262 to 1912 bp). Our findings reveal that the seven novel mitogenomes, exhibiting a nucleotide composition akin to that of typical insects, display a pronounced A + T bias, with their A + T content spanning from 75.61% in *Drusus annulatus* to 81.20% in *Moropsyche* sp. GX-2024 (refer to Table S3 for details). Notably, the CR exhibited the highest concentration of A + T content (92.75% to 89.01%), whereas PCGs show the lowest A + T content (72.59% to 79.54%). The A + T content of tRNA was lower than that of *l-rRNA* and *s-rRNA*. Among the PCGs, the A + T content was lowest at the second codon position and highest at the third codon position. The new mitogenomes of all species demonstrated positive A + T skew and negative G + C skew. Negative A + T skew and G + C skew were observed in the PCGs and CR, whereas positive A + T skew and G + C skew were found in the tRNAs and rRNAs. The skew analysis and nucleotide composition are presented in Table S3.

### 3.3. Codon Usage of PCGs

**Figure 2:**
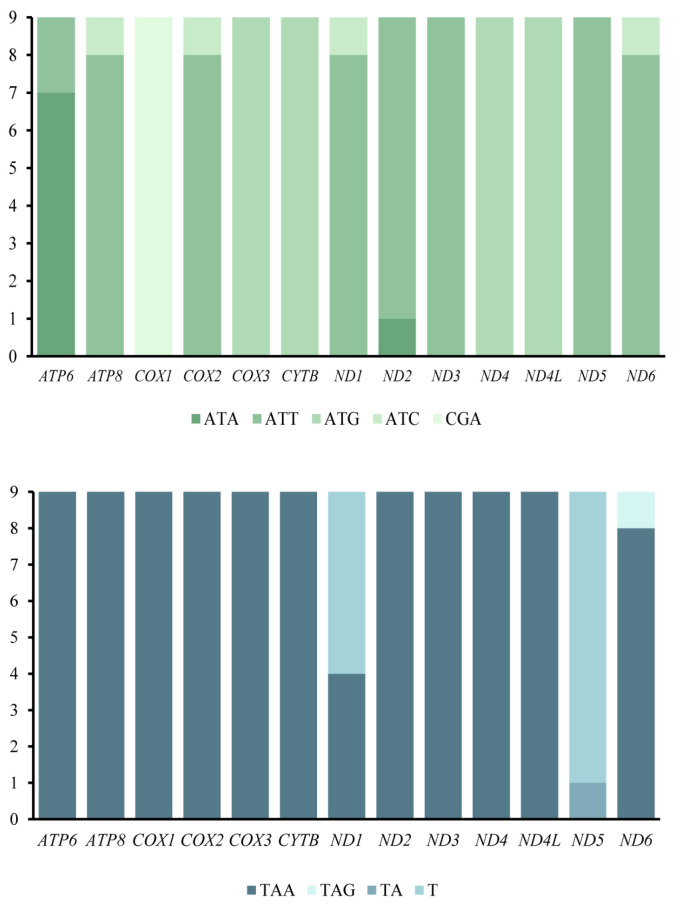
Start codons and end codons of PCGs among eight Apataniidae species and one Limnephlidae species generic complex mitogenomes.

**Figure 3:**
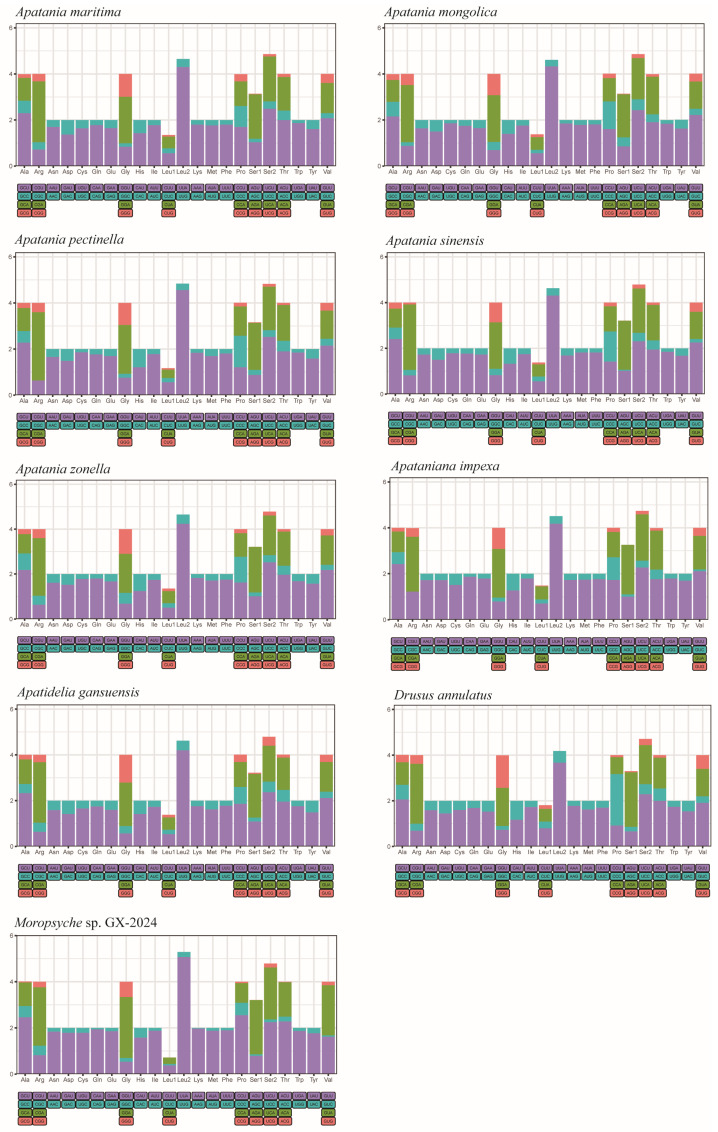
The relative synonymous codon usage (RSCU) of the mitochondrial PCGs of nine species. The X-axis shows different amino acids, and the Y-axis shows the RSCU value (the number of times a certain synonymous codon is used/the average number of times that all codons coding the amino acid are used).

Details of the start and stop codons of the nine new mitogenomes of 13 PCGs were shown in Figure 2. Except for the *COX1* gene, which has CGA as its start codon, the other PCGs exhibit start codons of the ATN pattern. The stop codons for the 13 PCGs were either the conventional TAA/TAG or the atypical T/TA. We analyzed the relative synonymous codon usage (RSCU) of the eight apataniid species of the one limnephilid species mitogenome of 13 PCGs for the first time (Figure 3). The results indicated that invertebrate 62 codons were used in mitogenomes of other species, except for the *Apatania pectinella*, *Apatania sinensis*, and *Moropsyche* sp. GX-2024 mitogenome, which each used only 61 codons due to the absence of AGG(S). The preferred codon in Apataniidae species is the AT enrichment codon UUA, and the frequency of utilization of UUA codons is more than 4% in *Apatania pectinella* and *Moropsyche* sp. GX-2024. In addition, the most frequently used amino acids are also AUU(Ile), AUA (Met), and UUU (Phe).

### 3.4. Substitution Rates and Nucleotide Diversity

**Figure 4:**
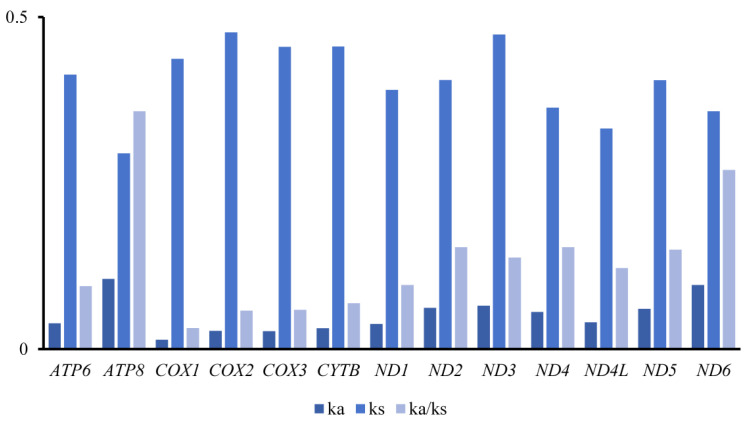
The evolution rate of 13 PCGs of Apataniidae. Ka refers to nonsynonymous nucleotide substitutions, Ks refers to synonymous nucleotide substitutions, and Ka/Ks refers to the selection pressure of each PCG. The abscissa represents the 13 PCGs, and the ordinate represents Ka/Ks values.

The average ratio of Ka to Ks substitution can be used to estimate the evolutionary rate and the degree of selection pressure of PCGs [41]. Our results showed that the substitution ratios of the 13 PCGs were all less than 1 and ranged from 0.36 (*ATP8*) to 0.03 (*COX1*) (Figure 4). All PCGs were undergoing purifying selection, with *COX1* being the strongest and *ND6* the weakest. This is similar to previous studies of the trichopteran mitogenome. The evolution rate of 13 PCGs was as follows: *ATP8* > *ND6* > *ND4* > *ND2* > *ND5* > *ND4L* > *ND3* > *ND1* > *ATP6* > *CYTB* > *COXIII* > *COXII* > *COXI*. By comparing the rate of evolution of other family within Limnephiloidea, we find that the selection pressure of the family with Limnephiloidea is higher than that of Hydropsychoidea and Philopotamoidea [21,24]. The nucleotide diversity of the 13 PCGs is highly variable in Apataniidae (Table S4). The highest Pi can be found in *ND1* (0.231), follow by the *ND6* (0.151) and *ND5* (0.139). and the lowest Pi for *ND4L* (0.094).

### 3.5. Mitochondrial Phylogenetic Analyses of Apataniidae

The aim of this study was to reconstruct phylogenetic relationships of Apataniidae. We therefore selected partly available species of Limnephiloidea in NCBI, and representative species mitogenomes available per subfamily Limnephilidae. The 13 PCGs and two rRNAs genes were aligned and trimmed, subsequently producing five matrices. The results include: (1) PCG12 matrix contained 7416 sites; (2) PCG matrix contained 11,124 sites; (3) PCG12R matrix contained 9519 sites; (4) PCG123R matrix contained 13,227 sites; (5) PCGAA matrix contained 3708 sites. For all of the datasets, the heterogeneity among the apataniid species was lower than that between them and other species of Limnephiloidea (Figure S1). The heterogeneity of sequence divergence of the PCGFAA dataset is the lowest, while the heterogeneity of PCG123R is the highest. Substitution saturation testing assessed the amount of phylogenetic effective site information contained in sequences. We used DAMBE to test the substitution saturation at different sites of PCGSs (codon1, codon2, and codon3). All of the results showed a lower ISS value than ISS.c value (*p* < 0.05), which indicated that they were not saturated (Figure S2). The matrices are feasible to use in phylogenetic reconstruction. The best model for each partition of the five matrixes was shown in the Table S5.

**Figure 5:**
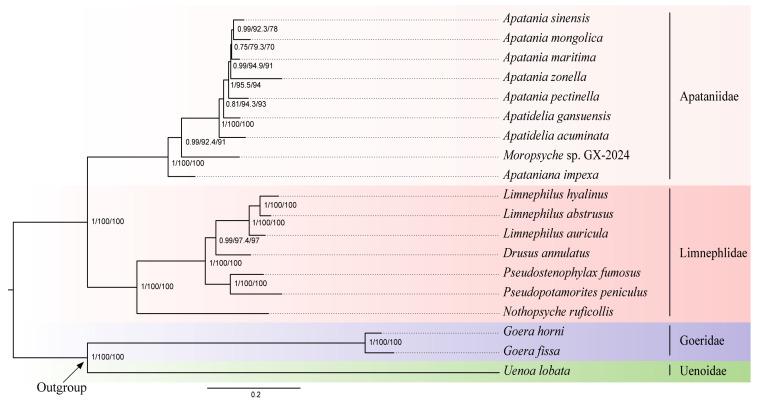
Phylogenetic tree generated based on the BI analysis of the PCGAA matrix dataset under the CAT + GTR model, and the ML analysis of the PCGAA matrix dataset. The numbers above the nodes are Bayesian posterior probabilities and bootstrap probabilities (SH-aLRT and UFBoot2).

In this study, five datasets (PCGFAA, PCG12, PCG, PCG12R, and PCG123R) were used to infer the phylogenetic relationships of Apataniidae through ML and BI methods, which generated different topologies. The monophyly of Apataniidae and Limnephilidae were highly supported. However, the monophyly of each subfamily were not well recovered within Apataniidae. The ML trees were reconstructed by the PCG12R and PCGFAA matrices, and the BI tree based on the PCG12R, PCGFAA, PCG123R, PCG12 consistently showed the topology: (*Apataniana* + (*Moropsyche* + (*Apatidelia* + *Apatania*))) (Figure 5 and Figures S3–S8). In the ML tree of PCG123R, four genera formed the topology of (*Moropsyche* + (*Apataniana* + (*Apatidelia* + *Apatania*))) (Figure S9). The ML analysis of PCG12 and PCG showed that (*Moropsyche* + *Apataniana*) + (*Apatidelia* + *Apatania*) (Figures S10 and S11), which were also found in the BI tree of the PCG matrix (Figure S12).

## 4. Discussion

### 4.1. Mitogenome Organization and Composition

To date, only one complete and one incomplete mitogenome of Apataniidae has been reported [22,23], which limits our understanding of the apataniid mitogenome. In this study, we successfully assembled the mitogenomes of eight species of Apataniidae and one species of Limnephilidae. The structural feature and nucleotide composition of all newly sequenced mitogenomes were similar to previously published mitogenome of Trichoptera and Limnephiloidea [23]. The gene order was the same in all studied mitogenomes, consistent with the gene arrangement of the ancestral insect mitogenome [42]. The length difference of the control region determines the length difference of the mitogenome [24]. All eight mitogenomes have obvious positive AT-skew and negative GC-skew, which may be related to the asymmetric mutation process in the replication process [43,44]. Codon usage analysis shows that Ile is the most common codon family. In addition, the start codon of *COX1* gene in eight mitogenomes is CGA, which confirms that CGA as the *COX1* start codon is a genic synapomorphy in Apataniidae. The results of non-synonymous and synonymous substitution ratio showed that all genes were undergoing strong purification selection, indicating a low evolution rate of mitogenome. Our nucleotide diversity results suggest that the *ND4L* were conserved genes in Apataniidae. Therefore, the *ND4L* may be an effective molecular marker for the classification of Apataniidae. The published mitogenome of Apataniidae also facilitates the application of multi-marker DNA metabarcoding in the rapid identification of aquatic insects [45].

### 4.2. Phylogenetic Relationships of Apataniidae

The phylogenetic relationship of Apataniidae has long been questionable. It has been shown that the family was divided into two subfamilies, but the phylogenetic position of some genera is uncertain, and they do not belong to any subfamily [46]. Previous studies on the phylogeny of Trichoptera based on molecular systematics have included only a small number of species from the family Apataniidae [47]. Moreover, monophyletic Apataniidae was not recovered in some studies, for example, the genus *Allomyia* formed a sister group with the genus *Archithremma* (Goeridae) [48,49].

In our study, all of the results strongly support monophyly and sister group relationships between Apataniidae and Limnephilidae. At the subfamily level, we obtained three topologies, and the classification status of two subfamilies (Apataniinae and Moropsychinae) was only recovered in the ML tree of the PCG123R matrix, but the clade was not well supported (Figure S8). In other topology, the genus *Apataniana* was recovered as a sister group to *Moropsyche*, but it had lower nodal support values, while this topology is well supported when *Apataniana* was located at the base of Apataniidae. Therefore, whether *Apataniana* belongs to Apataniinae needs to be further researched. In addition, the phylogenetic analyses based on three topologies revealed that *Apatidelia* was a non-monophyletic group; in other words, *Apatania* and *Apatidelia* form a paraphyletic group. Previous studies have shown that *Apatania* and *Apatidelia* are sister lineages. Compared with *Apatania*, the adult sternum V of *Apatidelia* with lateral lobes is a unique apomorphy [50]. However, we examined *Apatidelia gansuensis* and found that lateral lobes of sternum V was not obvious. Therefore, we believe that the monophyly of *Apatidelia* needs to be confirmed by more evidence. To better understand the phylogenetic relationships of *Apatania*, *Apataniana*, and *Apatidelia*, the revision of three genera relationship should be conducted first. Then, more phylogenomic data and better taxon sampling are required to resolve phylogenetic relationships at the generic or even subfamily level.

## 5. Conclusions

This study provides the mitogenomes of *Apatania*, *Apataniana*, and *Moropsyche*, three genera in Apataniidae, for the first time. Comparative analyses indicate that the apataniid mitogenomes are structurally conserved and exhibit putative ancestral gene order. The nucleotide composition of the nine mitogenomes is distinctly A + T biased. The CGA as the *COX1* start codon is a synapomorphy in Apataniidae. Evolutionary rates vary among species, and the length and nucleotide composition in the control region are highly variable as well. The *ND4L* may be an effective molecular marker for the classification of the Apataniidae. The phylogenetic analyses based on mitogenome for the first time recovers the monophyly of Apataniidae, and the sister group relationship is Limnephilidae. Except for PCG, BI tree based on other matrices consistently showed the topology: (*Apataniana* + (*Moropsyche* + (*Apatidelia* + *Apatania*))). *Apatania* and *Apatidelia* form a paraphyletic group, and the monophyly of *Apatidelia* needs to be confirmed by more evidence.

## Data Availability

The voucher specimens from this research were deposited in the College of Plant Protection, Nanjing Agricultural University, Nanjing, China, and the College of Life Science and Technology, Tianjin normal University, Tianjin, China. The newly sequenced mitochondrial genome in this study has been uploaded to GenBank (PQ568869-PQ568877).

## Supplementary Materials

The following supporting information can be downloaded at: https://www.mdpi.com/article/10.3390/insects15120973/s1, Table S1: Collection information of the newly sequenced samples; Table S2: Detailed taxonomic resources used in present study; Table S3: Nucleotide composition of nine mitogenomes; Table S4: Nucleotide diversity (Pi) values of nine apataniid species of PCGs; Table S5: Final gene partitions for the Maximum Likelihood Phylogenetic analysis; Figure S1: The assessment of the heterogeneity among the mitogenomes of five matrixes focused on their PCGs and rRNAs. The degree of sequence similarity was visually represented through colored blocks, utilizing AliGROOVE scores that span a spectrum from −1 (indicating substantial heterogeneity between matrixes, represented by red) to +1 (signaling minimal heterogeneity between matrixes, depicted in blue). (A) PCG. (B) PCG12. (C) PCG12R. (D) PCGAA. (E) PCG123R; Figure S2: Substitution saturation plots of PCGs for matrix. Plots in blue and green indicate transition and transversion, respectively; Figure S3: Phylogenetic tree generated based on the BI analysis of the PCG12 matrix dataset under the CAT + GTR model. The numbers above nodes are Bayesian posterior probabilities; Figure S4: Phylogenetic tree generated based on the ML analysis of the PCGAA matrix dataset under the Partitioning model. The numbers above nodes are bootstrap probabilities; Figure S5: Phylogenetic tree generated based on the ML analysis of the PCGAA matrix dataset under the PMSF model. The numbers above the nodes are bootstrap probabilities; Figure S6: Phylogenetic tree generated based on the BI analysis of the PCG123R matrix dataset under the CAT + GTR model. The numbers above the nodes are Bayesian posterior probabilities; Figure S7: Phylogenetic tree generated based on the ML analysis of PCG12R matrix with Partitioning model. The numbers above the nodes are bootstrap probabilities; Figure S8: Phylogenetic tree generated based on the BI analysis of the PCG12R matrix dataset under the CAT + GTR model. The numbers above the nodes are Bayesian posterior probabilities; Figure S9: Phylogenetic tree generated based on the ML analysis of the PCG123R matrix dataset under the Partitioning model. The numbers above the nodes are bootstrap probabilities; Figure S10: Phylogenetic tree generated based on the ML analysis of PCG12 matrix with Partitioning model. The numbers above the nodes are bootstrap probabilities; Figure S11: Phylogenetic tree generated based on the ML analysis of the PCG matrix dataset under the Partitioning model. The numbers above the nodes are bootstrap probabilities; Figure S12: Phylogenetic tree generated based on the BI analysis of the PCG matrix dataset under the CAT + GTR model. The numbers above the nodes are Bayesian posterior probabilities.
