# Supplementary material for: Mitogenomics Provide New Phylogenetic Insights of the Family Apataniidae (Trichoptera: Integripalpia)
Source: Insects. 2024 Dec 6;15(12):973. doi: 10.3390/insects15120973 (PMC11677691; doi:10.3390/insects15120973)
Supplement: Supplementary file 1 [file insects-15-00973-s001.zip › insects-3338121-supplementary.pdf]

Table S1. Collection information of the newly sequenced samples.

Species	Geographical locality	Longitude	Latitude	Elevation (m)	Collection date
Moropsyche sp. GX-2024	Guilin, Guangxi, China	109.9420°E	25.5593°N,	1,350.1	18-VI-2023
Apatidelia gansuensis	Menyuan, Qinghai, China	101.8467°E	37.2996°N	2,678.3	2-VII-2021
Apataniana impexa	Delingha, Qinghai, China	97.2570°E	37.6306°N	4,126.1	16-VII-2021
Apatania sinensis	Taoyuan, Hunan, China	110.7723°E	29.9414°N	253.3	6-III-2021
Apatania pectinella	Taoyuan, Hunan, China	110.7723°E	29.9414°N	253.3	6-III-2021
Apatania mongolica	Menyuan, Qinghai, China	101.6081°E	37.3526°N	2,786.5	29-VII-2020
Apatania maritima	Yili, Xinjiang, China	81.3698°E	44.6113°N	2,065.3	2-V-2019
Apatania zonella	Haidong, Xinjiang, China	89.0303°E	43.779779°N	1,681.3	24-VI-2024

Table S2. Detailed taxonomic resources used in present study.

Family	Genus	Species	GenBank access
Apataniidae	*Moropsyche*	*Moropsyche* sp. GX-2024	PQ568877
	Apatidelia	*Apatidelia acuminata* Leng & Yang, 1998	OL678002
		Apatidelia gansuensis Mey, 1997	PQ568875
	Apataniana	*Apataniana impexa* Schmid, 1968	PQ568873
	Apatania	*Apatania sinensis* Martynov, 1914	PQ568872
		Apatania pectinella Mey & Yang, 2001	PQ568870
		Apatania mongolica Martynov, 1914	PQ568871
		Apatania maritima Ivanov & Levanidova, 1993	PQ568869
		Apatania zonella Zetterstedt, 1840	PQ568877
Limnephlidae	*Drusus*	*Drusus annulatus* Stephens, 1837	PQ568875
	Limnephilus	*Limnephilus hyalinus* Hagen, 1861	MK077681
		Limnephilus abstrusus McLachlan, 1872	OL678028
		Limnephilus auricula Curtis, 1834	OX638418
	Nothopsyche	*Nothopsyche ruficollis* Ulmer, 1905	OL678035
	Pseudostenophylax	*Pseudostenophylax fumosus* Martynov, 1909	OL678045
	Pseudopotamorites	*Pseudopotamorites peniculus* Forsslund, 1935	OL678044
Goeridae	*Goera*	*Goera horni* Navas, 1926	OL678017
		Goera fissa Ulmer, 1926	OL678016
Uenoidae	*Uenoa*	*Uenoa lobata* Hwang, 1957	OL678055

Table S3. Nucleotide composition of 9 mitogenomes

Regions	Species	Whole genome	PCGs	Site 1	Site 2	Site 3	tRNA	l-rRNA	s-rRNA	CR
length(bp)	*Moropsyche* sp. GX-2024	15,070	11,220	3,741	3,738	3,738	1,453	1,364	785	262
	Apatidelia gansuensis	15,509	11,215	3,739	3,736	3,736	1,452	1,364	782	687
	Apataniana impexa	15,306	11,205	3,736	3,733	3,733	1,464	1,388	786	399
	Apatania sinensis	15,398	11,216	3,740	3,738	3,738	1,449	1,389	780	556
	Apatania pectinella	15,557	11,218	3,740	3,738	3,737	1,450	1,388	787	710
	Apatania mongolica	15,435	11,216	3,740	3,738	3,738	1,448	1,375	782	597
	Apatania maritima	16,737	11,220	3,741	3,738	3,738	1,453	1,369	782	1,912
	Apatania zonella	15,984	11,219	3,741	3,739	3,739	1,451	1,385	778	1,136
	Drusus annulatus	15,299	11,187	3,731	3,728	3.728	1,444	1,373	787	546
A+T%	*Moropsyche* sp. GX-2024	81.20	79.54	75.30	70.79	92.54	83.96	86.80	85.22	92.75
	Apatidelia gansuensis	77.94	75.10	72.10	70.10	83.08	82.44	85.26	85.17	91.27
	Apataniana impexa	78.74	76.42	72.03	70.16	87.09	82.38	85.23	85.62	89.47

	Apatania sinensis	78.73	76.27	72.33	70.14	86.36	83.23	85.31	85.26	89.21
	Apatania pectinella	78.80	76.30	72.83	70.39	85.68	83.45	84.87	85.39	88.73
	Apatania mongolica	78.30	75.86	72.03	70.12	85.42	82.60	85.09	84.53	89.28
	Apatania maritima	79.49	76.13	72.15	70.17	86.06	83.07	84.81	85.55	89.96
	Apatania zonella	78.52	75.39	71.88	70.34	83.95	83.18	85.13	85.09	90.23
	Drusus annulatus	75.61	72.59	69.07	68.80	79.91	80.82	84.20	83.99	89.01
G+C%	*Moropsyche* sp. GX-2024	18.80	20.46	24.70	29.21	7.46	16.04	13.20	14.78	7.25
	Apatidelia gansuensis	22.06	24.90	27.90	29.90	16.92	17.56	14.74	14.83	8.73
	Apataniana impexa	21.25	23.58	27.97	29.84	12.91	17.62	14.77	14.38	10.28
	Apatania sinensis	21.27	23.73	27.67	29.86	13.64	16.77	14.69	14.74	10.79
	Apatania pectinella	21.20	23.70	27.17	29.61	14.32	16.55	15.13	14.61	11.27
	Apatania mongolica	21.69	24.14	27.97	29.88	14.58	17.40	14.91	15.47	10.55
	Apatania maritima	20.50	23.87	27.85	29.83	13.94	16.93	15.19	14.45	9.99
	Apatania zonella	21.47	24.60	28.09	29.66	16.05	16.82	14.87	14.91	9.77

	Drusus annulatus	24.39	27.41	30.93	31.20	20.09	19.18	15.80	16.01	10.99
AT-Skew	*Moropsyche* sp. GX-2024	0.00	-0.12	0.03	-0.38	-0.04	0.04	0.04	0.07	-0.09
	Apatidelia gansuensis	0.02	-0.13	0.05	-0.39	-0.08	0.02	0.03	0.05	-0.05
	Apataniana impexa	0.03	-0.13	0.05	-0.39	-0.07	0.03	0.01	0.03	-0.12
	Apatania sinensis	0.02	-0.13	0.05	-0.39	-0.06	0.02	0.02	0.04	-0.06
	Apatania pectinella	0.03	-0.13	0.04	-0.39	-0.06	0.03	0.01	0.02	-0.09
	Apatania mongolica	0.02	-0.13	0.05	-0.39	-0.07	0.02	0.01	0.03	-0.08
	Apatania maritima	0.01	-0.13	0.05	-0.39	-0.07	0.03	0.03	0.04	-0.06
	Apatania zonella	0.02	-0.13	0.05	-0.39	-0.07	0.03	0.03	0.04	-0.05
	Drusus annulatus	0.03	-0.14	0.04	-0.40	-0.07	0.03	-0.01	0.01	-0.01
GC-Skew	*Moropsyche* sp. GX-2024	-0.21	-0.01	0.19	-0.12	-0.28	0.10	0.30	0.24	-0.05
	Apatidelia gansuensis	-0.24	-0.02	0.17	-0.12	-0.16	0.10	0.28	0.28	-0.27
	Apataniana impexa	-0.24	-0.04	0.13	-0.12	-0.22	0.10	0.25	0.27	-0.02
	Apatania sinensis	-0.24	-0.04	0.17	-0.13	-0.26	0.14	0.29	0.22	-0.40

	Apatania pectinella	-0.24	-0.04	0.19	-0.13	-0.27	0.11	0.30	0.29	-0.25
	Apatania mongolica	-0.22	-0.05	0.16	-0.14	-0.29	0.13	0.28	0.29	-0.21
	Apatania maritima	-0.23	-0.03	0.17	-0.14	-0.22	0.09	0.25	0.22	-0.29
	Apatania zonella	-0.22	-0.04	0.17	-0.13	-0.25	0.10	0.27	0.24	-0.24
	Drusus annulatus	-0.27	-3.26	0.14	-0.11	-0.18	0.08	0.33	0.30	-0.20

Table S4. Nucleotide diversity (Pi) values of nine apataniid species of PCGs.

Gene	Pi
ATP6	0.12378
ATP8	0.13889
COX1	0.11349
COX1	0.11623
COX1	0.12337
CYTB	0.12555
ND1	0.23085
ND2	0.13265
ND3	0.13826
ND4	0.12424
ND4L	0.09383
ND5	0.13928
ND6	0.15107

Table S5. Final gene partitions for the Maximum Likelihood Phylogenetic analysis

Matrixs	Partition names	Best model
PCGAA matrix	*ATP6, COX1, COX2, COX3, CYTB, ND3*	mtMet+R3
	ATP8, ND2, ND6	mtMet+F+G4
	ND1, ND4, ND4L, ND5	mtInv+R4
PCG matrix	*ATP6, ND2, ND3, ND6*	GTR+F+R3
	ATP8, COX1, COX2, COX3, CYTB	GTR+F+R4
	ND1, ND4L	TVM+F+R3
	ND4, ND5	TVM+F+I+G4
PCG123R matrix	*ATP6, ND2, ND3, ND6*	GTR+F+I+G4
	ATP8, COX1, COX2, COX3, CYTB	GTR+F+R4
	ND1, ND4L, l-rRNA, s-rRNA	TVM+F+R3
	ND4, ND5	TVM+F+I+G4
PCG12 matrix	*ATP6, COX2, COX3, CYTB*	TIM2+F+I+G4
	ATP8, ND2, ND3, ND6	GTR+F+R3
	COX1	TIM2+F+R2
	ND1, ND4L	TVM+F+R2
	ND4, ND5	GTR+F+I+G4
PCG12R matrix	*ATP6, COX2, COX3, CYTB*	TIM2+F+I+G4
	ATP8, ND2, ND3, ND6	GTR+F+R3
	COX1	TIM2+F+R2
	ND1, ND4L	TVM+F+R2
	ND4, ND5	GTR+F+I+G4
	l-rRNA, s-rRNA	TVM+F+R3

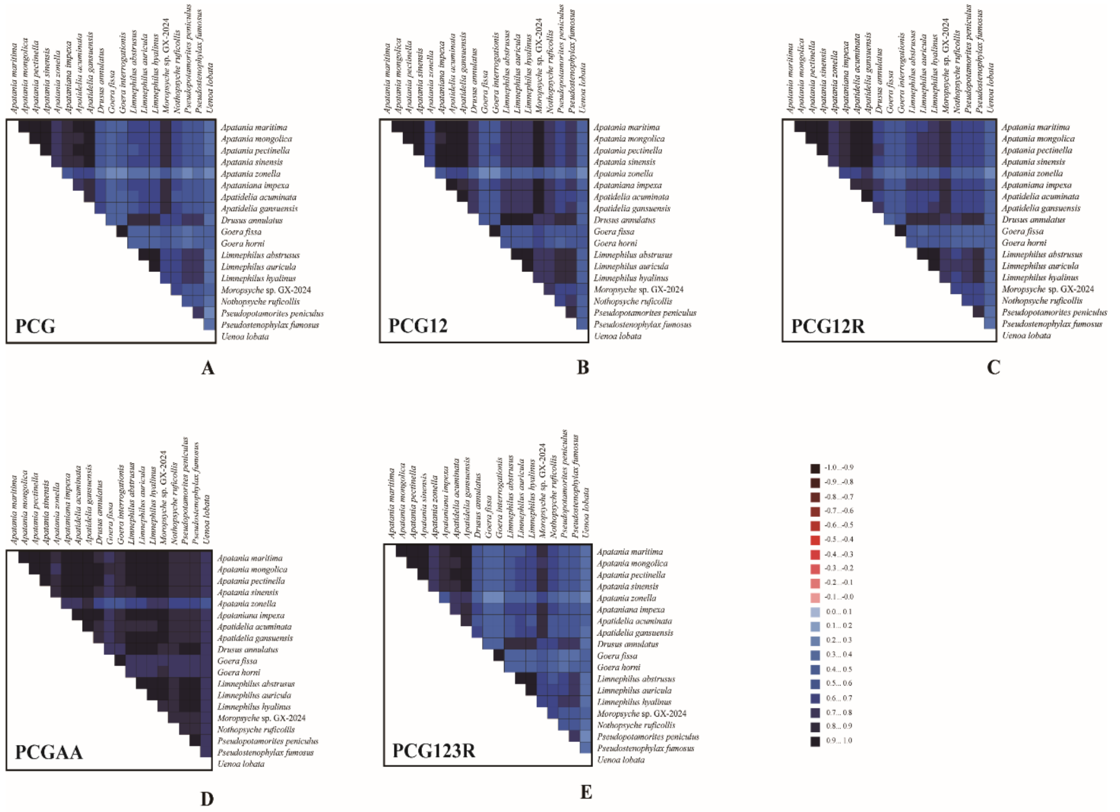

Figure S1. The assessment of the heterogeneity among the mitogenomes of five matrixes focused on their PCGs and rRNAs. The degree of sequence similarity was visually represented through colored blocks, utilizing AliGROOVE scores that span a spectrum from −1 (indicating substantial heterogeneity between matrixes, represented by red) to +1 (signaling minimal heterogeneity between matrixes, depicted in blue). (A) PCG. (B) PCG12. (C) PCG12R. (D) PCGAA. (E) PCG123R.

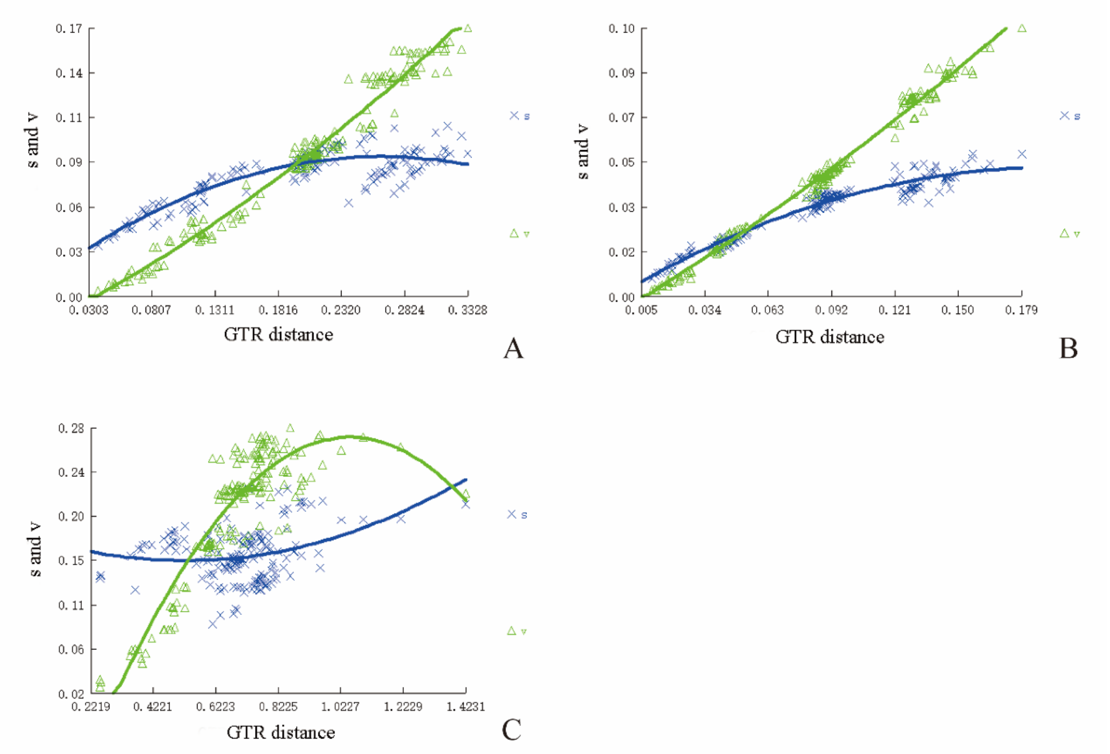

Figure S2 Substitution saturation plots of mt genomic PCGs for 9 species. Plots in blue and green indicate transition and transversion, respectively. (**A**) First codon position (**B**) Second codon position (**C**) Third codon position.

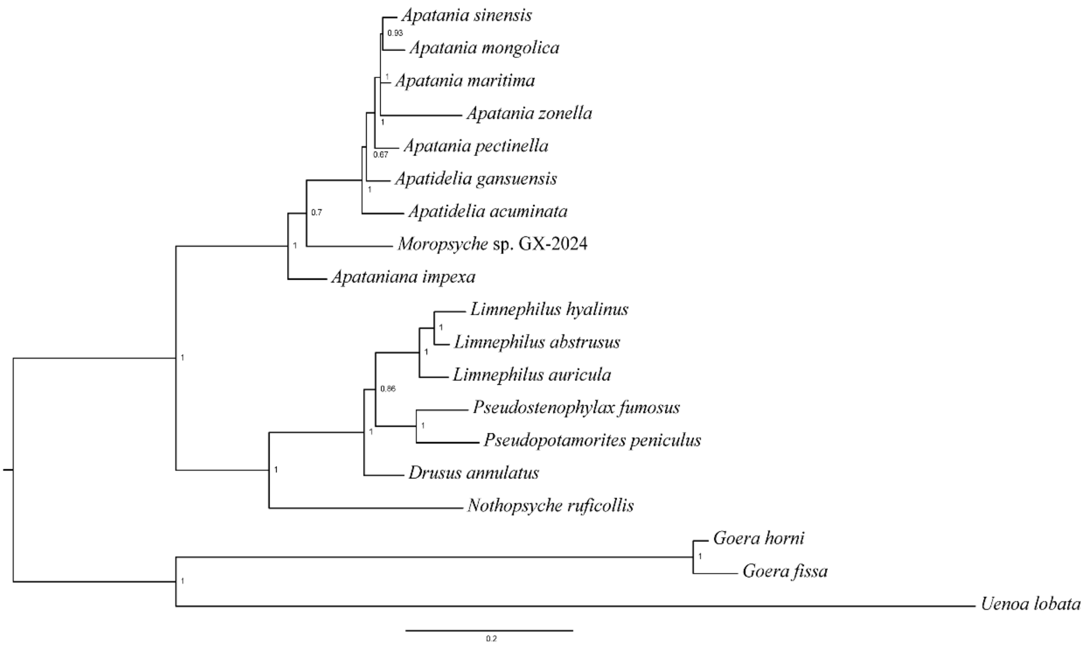

Figure S3. Phylogenetic tree generated based on the BI analysis of the PCG12 matrix dataset under the CAT+GTR model. The numbers above nodes are Bayesian posterior probabilities.

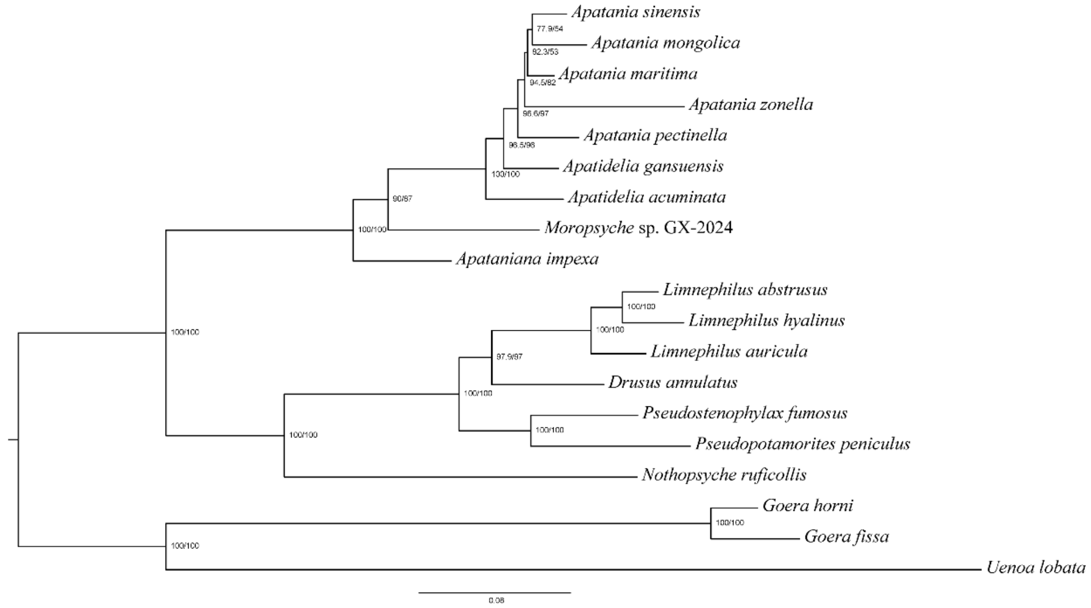

Figure S4. Phylogenetic tree generated based on the ML analysis of the PCGAA matrix dataset under the Partitioning model. The numbers above nodes are bootstrap probabilities.

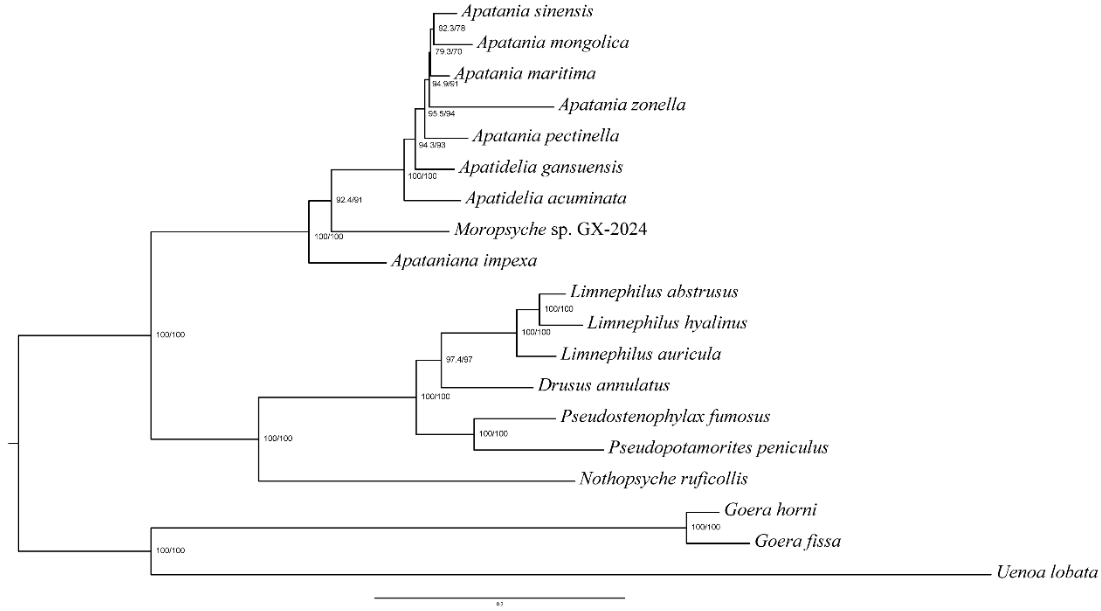

Figure S5. Phylogenetic tree generated based on the ML analysis of the PCGAA matrix dataset under the PMSF model. The numbers above the nodes are bootstrap probabilities.

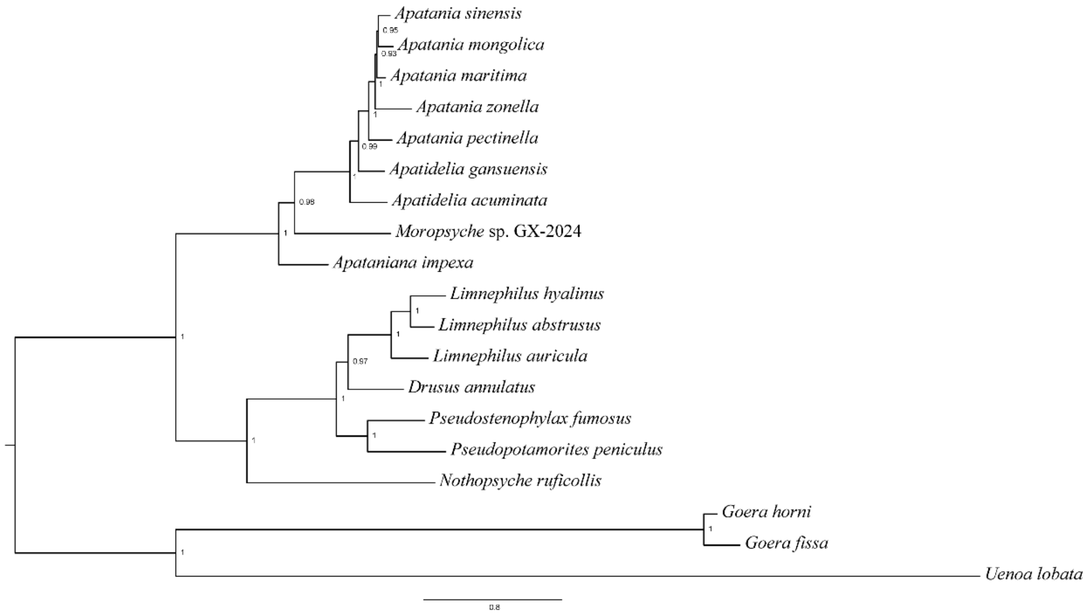

Figure S6. Phylogenetic tree generated based on the BI analysis of the PCG123R matrix dataset under the CAT + GTR model. The numbers above the nodes are Bayesian posterior probabilities.

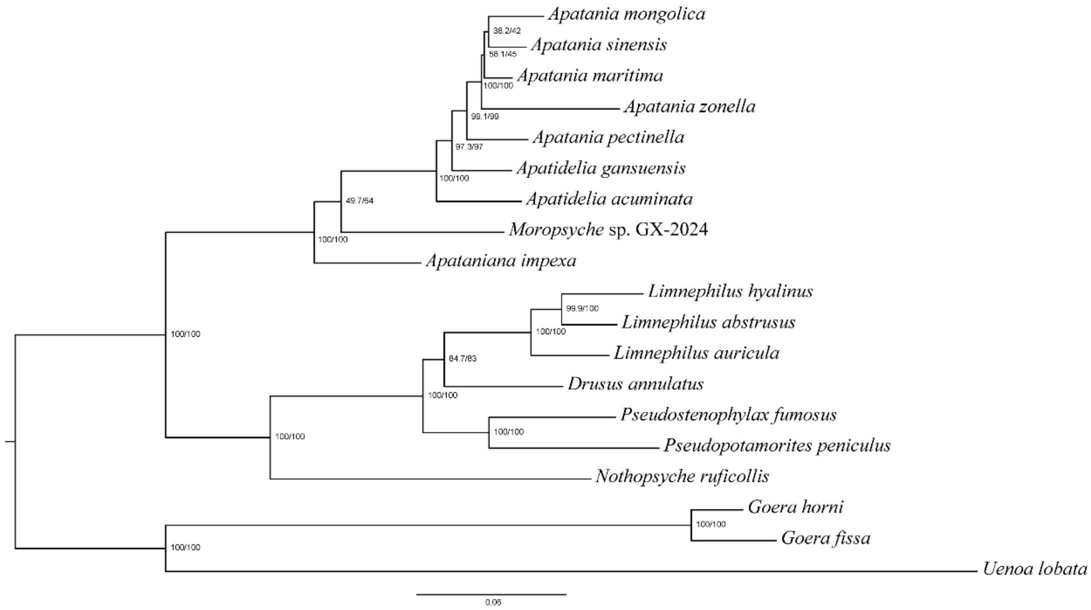

Figure S7. Phylogenetic tree generated based on the ML analysis of PCG12R matrix with Partitioning model. The numbers above the nodes are bootstrap probabilities.

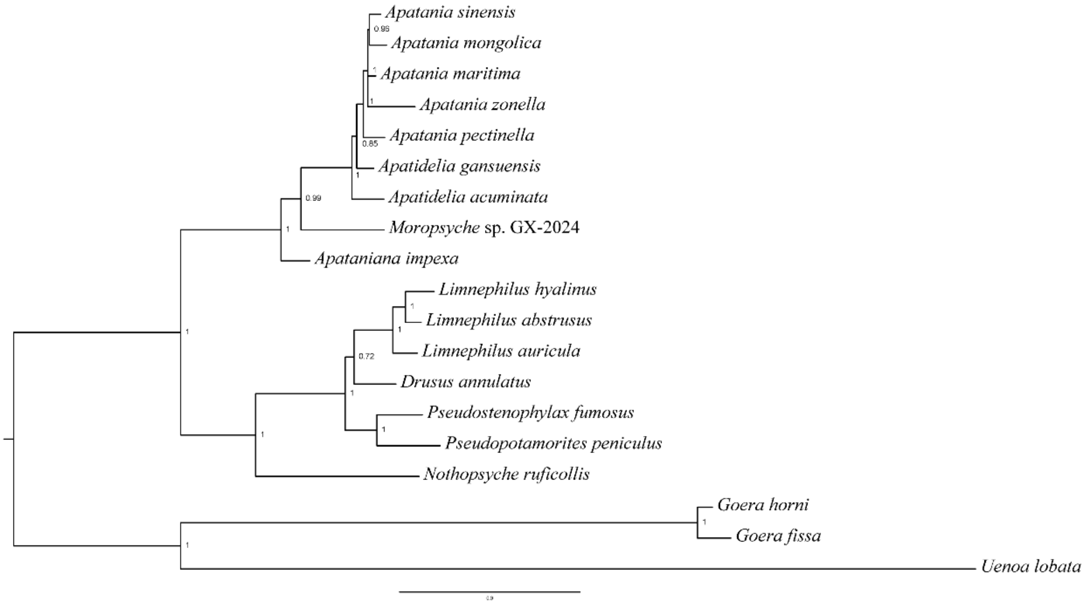

Figure S8. Phylogenetic tree generated based on the BI analysis of the PCG12R matrix dataset under the CAT+GTR model. The numbers above the nodes are Bayesian posterior probabilities.

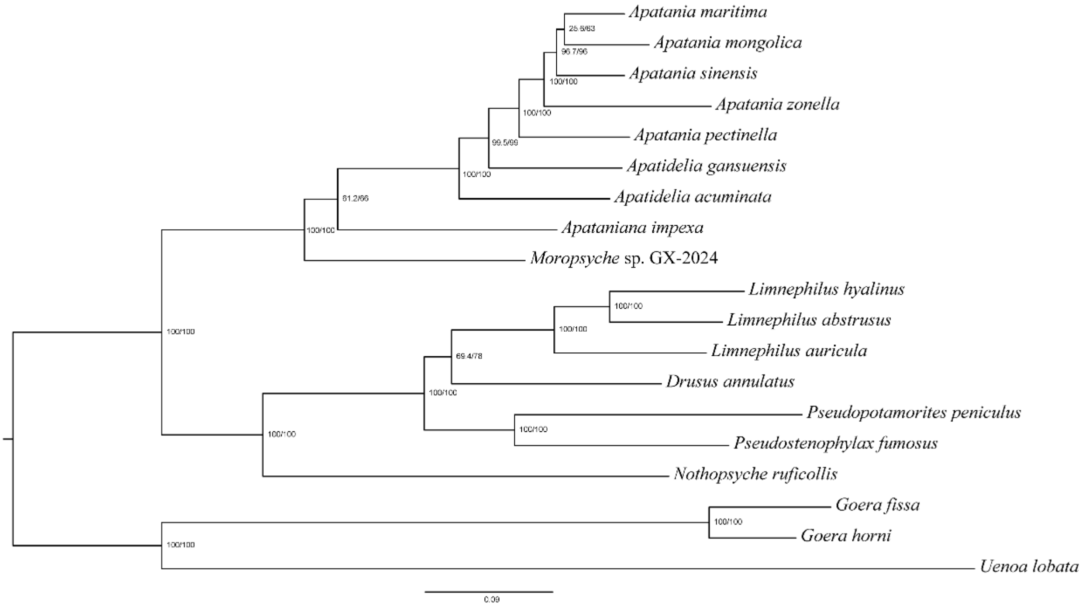

Figure S9. Phylogenetic tree generated based on the ML analysis of the PCG123R matrix dataset under the Partitioning model. The numbers above the nodes are bootstrap probabilities.

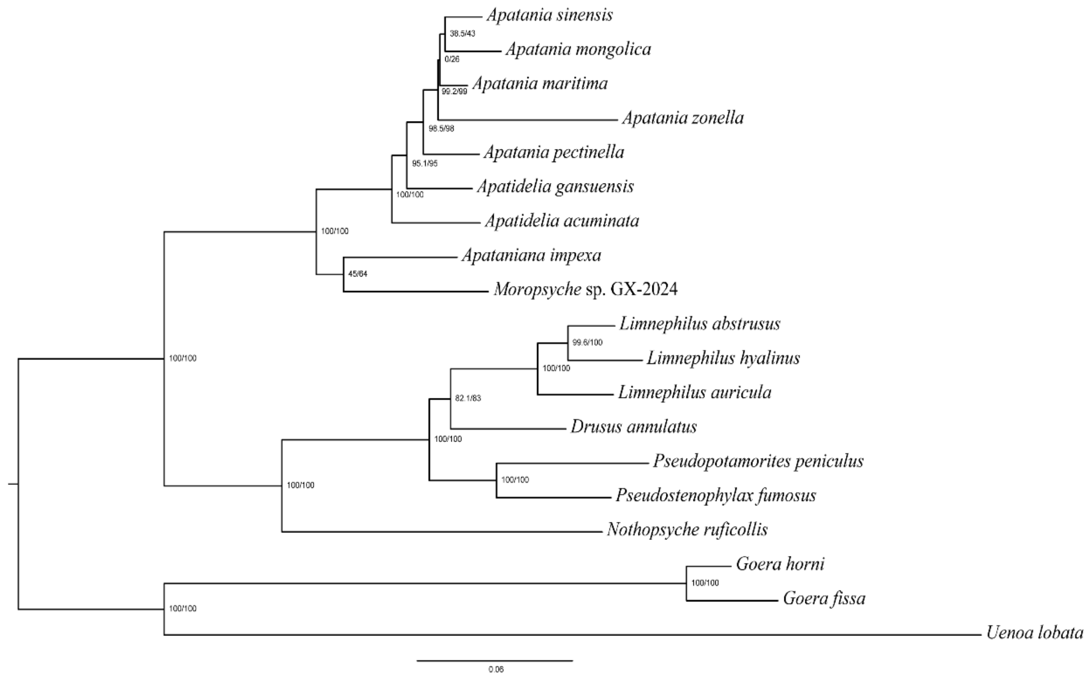

Figure S10. Phylogenetic tree generated based on the ML analysis of PCG12 matrix with Partitioning model. The numbers above the nodes are bootstrap probabilities.

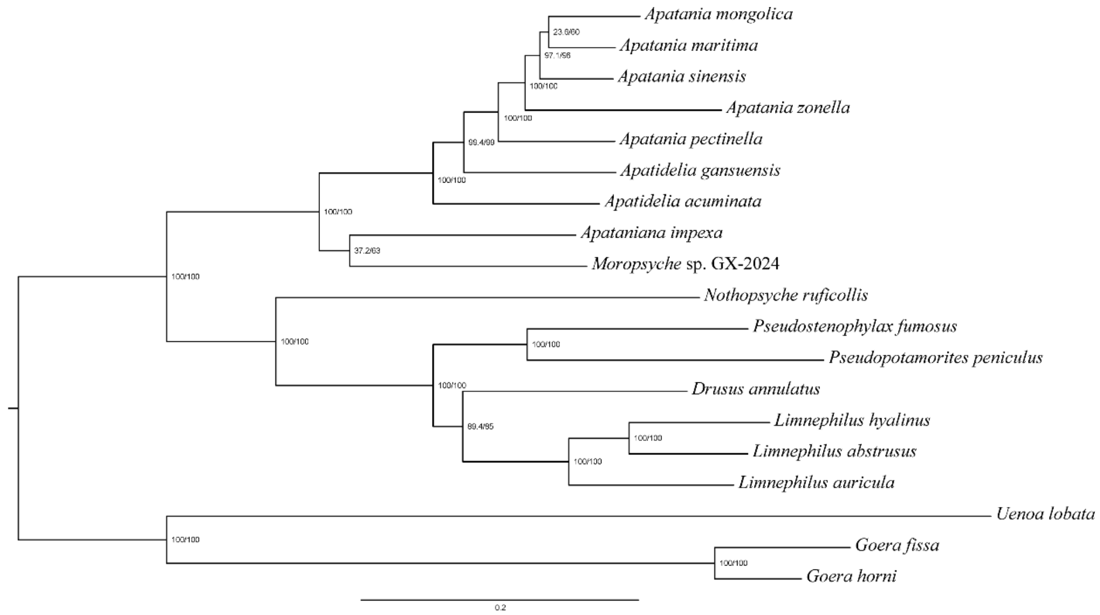

Figure S11. Phylogenetic tree generated based on the ML analysis of the PCG matrix dataset under the Partitioning model. The numbers above the nodes are bootstrap probabilities.

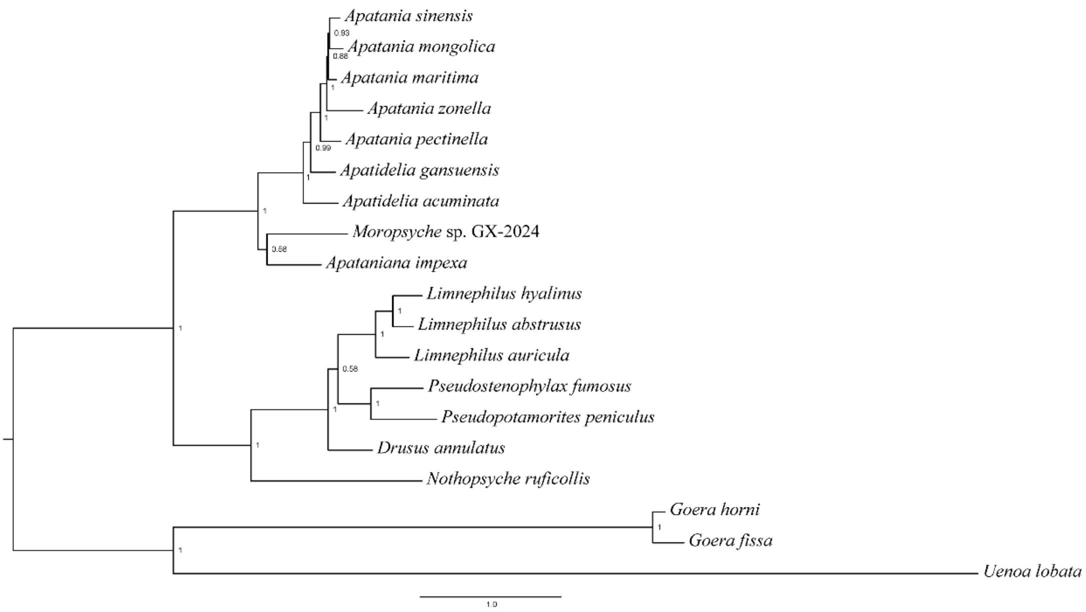

Figure S12. Phylogenetic tree generated based on the BI analysis of the PCG matrix dataset under the CAT + GTR model. The numbers above the nodes are Bayesian posterior probabilities.
